# Proposing novel natural compounds against Alzheimer’s disease targeting acetylcholinesterase

**DOI:** 10.1371/journal.pone.0284994

**Published:** 2023-04-27

**Authors:** Münteha Girgin, Sevim Isik, Nigar Kantarci-Carsibasi

**Affiliations:** 1 Department of Chemical Engineering, Uskudar University, Uskudar, Istanbul, Turkey; 2 Department of Molecular Biology and Genetics, Uskudar University, Uskudar, Istanbul, Turkey; 3 Uskudar University, Stem Cell Research and Application Center (USKOKMER), Istanbul, Turkey; Ahram Canadian University, EGYPT

## Abstract

Alzheimer’s disease (AD) is a neurodegenerative disorder considered as a global public health threat influencing many people. Despite the concerning rise in the affected population, there is still a shortage of potent and safe therapeutic agents. The aim of this research is to discover novel natural source molecules with high therapeutic effects, stability and less toxicity for the treatment of AD, specifically targeting acetylcholinesterase (AChE). This research can be divided into two steps: *in silico* search for molecules by systematic simulations and *in vitro* experimental validations. We identified five leading compounds, namely Queuine, Etoperidone, Thiamine, Ademetionine and Tetrahydrofolic acid by screening natural molecule database, conducting molecular docking and druggability evaluations. Stability of the complexes were investigated by Molecular Dynamics simulations and free energy calculations were conducted by Molecular Mechanics Generalized Born Surface Area method. All five complexes were stable within the binding catalytic site (CAS) of AChE, with the exception of Queuine which remains stable on the peripheral site (PAS). On the other hand Etoperidone both interacts with CAS and PAS sites showing dual binding properties. Binding free energy values of Queuine and Etoperidone were -71.9 and -91.0 kcal/mol respectively, being comparable to control molecules Galantamine (-71.3 kcal/mol) and Donepezil (-80.9 kcal/mol). Computational results were validated through in vitro experiments using the SH-SY5Y(neuroblastoma) cell line with Real Time Cell Analysis (RTCA) and cell viability assays. The results showed that the selected doses were effective with half inhibitory concentrations estimated to be: Queuine (IC50 = 70,90 μM), Etoperidone (IC_50_ = 712,80 μM), Thiamine (IC_50_ = 18780,34 μM), Galantamine (IC_50_ = 556,01 μM) and Donepezil (IC_50_ = 222,23 μM), respectively. The promising results for these molecules suggest the development of the next step *in vivo* animal testing and provide hope for natural therapeutic aids in the treatment of AD.

## Introduction

Alzheimer’s disease (AD) was first determined by Alois Alzheimer in 1907 and characterized as a long-term progressive neurodegenerative disorder of the central nervous system (CNS) and at present it is recognized as a global public health threat by the World Health Organization. AD is the most prevalent and widespread form of dementia observed worldwide. Although it has been 115 years since its discovery, the exact cause of AD is still unknown and there is still no definite cure. AD is determined by several clinical symptoms that include a progressive decline in memory, thinking, speech, learning capacities and resulting in behavioral abnormalities and unfortunately increases by twofold every five years in people beyond age 65 [1–3]. Although it is known that currently there are about 50 million ADs in the world, these numbers will continue to increase in the coming years and it is expected to reach 152 million by 2050 [4, 5].

In spite of an alarming increase in the population suffering from AD worldwide, there is a lack of effective therapeutic agents. Currently, there are several FDA-approved drugs used in the treatment of AD on the market such as donepezil, galantamine, rivastigmine, and memantine. However, none of these FDA-approved drugs could show sufficient effect and improvement in Alzheimer’s patients, i.e. they could not go beyond 50% success in reducing symptoms and delaying progression in early-stage AD, and showed almost no success in advanced AD cases. In addition, these approved drugs on the market cause serious side effects due to their limited therapeutic effects, non-target-specificity, low bioavailability and high hepatotoxicity values. For example, Tacrine, the first FDA-approved AChE-targeted drug, was removed from the market due to serious hepatotoxicity. Therefore, there is an urgent need for search of effective disease therapeutic interventions to cure AD through different approaches such as drug design [6]. For this purpose, much effort has been given to identify and study potential AD drugs, recently [1, 7–17].

Initial drug development strategies were usually based on the studies related to the pathogenesis of the disease. AD is pathologically related to several hypotheses such as the accumulation of amyloid beta (Aβ) plaques (amyloid cascade hypothesis), degeneration of cholinergic neurons in the absence of acetylcholine (ACh) neurotransmitter (cholinergic hypothesis), tau protein hypothesis, glutamergic cascade, lipid metabolism disorder hypothesis. Amongst these hypotheses, cholinergic hypothesis has become the most generally accepted hypothesis [18, 19] and the enzyme acetylcholinesterase (AChE), which is responsible for the degradation of ACh to cholin and acetate, has been an important biological drug target [15, 20–22]. Studies aimed to decrease AChE activity with a potent AChE inhibitor to be developed, and in this way increase in ACh concentration in the synapse which would lead to increased cholinergic transmission and hence diminish AD symptoms. Studies targeting acetylcholinesterase inhibition started around 1980’s. Since then, a wide range of evidence shows that AChE inhibitors can improve cognitive function and slow down the progression of AD [11, 23, 24].

AChE binding gorge is approximately 20 Å deep and has an ellipsoidal shape that broadens at the base where ACh hydrolysis takes place. This active site contains several subsites such as Catalytic Active Site (CAS site) at the base of the pocket, consisting of residues Ser203, Glu 334 and His 447; Peripheral Anionic Site (PAS) comprising of Asp 72, Asp74, Tyr124, Ser 125, Trp 286, Tyr 337, and Tyr 341; oxyanion hole formed by Gly 121, Gly 122, and Ala 204; anionic subsite lined with residues Trp 86, Tyr 133, Glu 202, Gly 448, and Ile 451; and finally acyl binding pocket comprised of Phe 295, Trp 236, Phe 297, Phe 338, Gly 122). It was reported that PAS is responsible for guiding the ligands to the binding site and recent studies showed a correlation of PAS in A*β* aggregation process which is one of the accepted hypotheses (amyloid cascade) observed in AD. It was also suggested that potent PAS inhibitors that will hinder the entry to AChE gorge would prevent the A*β* peptide binding to AChE, and in this way help to slow down the progression of AD [25, 26]. In this respect, dual binding AChE inhibitors which would interact with both CAS and PAS are emerging as an important research focus in AD treatment [6, 11].

Finding and synthesizing a novel drug and making it marketable requires a long process and high investments. While this process requires an average of 10–15 years and an investment cost of 1–3 billion dollars, the success rates are around 2% [27]. However, Covid-19 pandemic, which we have been exposed to since 2019 together with the whole world, demonstrated the necessity for urgent and effective remedy and treatment of diseases by drugs, vaccines, etc. in a very short time. In this respect, drug repurposing provides much more advantages in terms of both time and economy compared to de nevo (conventional) drug development methods. While researchers need 3–5 years to reposition the drug; research and development cost is around 0.3 billion dollars. The fact that comprehensive information about the formulations of the drug already exists, potential toxicity, safety and adverse drug reaction studies have already been carried out, greatly reduces the possibility of failure in terms of safety [28]. In the light of these data, drug development time is brought forward by 7–8 years with drug repositioning methods, while the costs are 10% of traditional new drug development [29, 30].

This work can be divided into two steps: *in silico* systematic simulations followed by *in vitro* experimental validation. Basically, the first step involves a repurposing strategy aiming to propose natural AChE inhibitors that may safely substitute the synthetic approved AD drugs. We aim to discover novel natural source molecules targeting AChE that have not been mentioned in AD treatment until now. We conducted systematic simulations including virtual screening, natural database generation, molecular docking, molecular dynamics, MM-GBSA binding free energy calculations and ADMET analysis. We proposed five hit natural molecules namely Ademetionine, Tetrahydrofolic acid, Thiamine, Queuine and Etoperidone which interact with important binding sites, specially dual binding properties to CAS and PAS of AChE by computational approaches. Among them, Folic acid, Ademetionine and Thiamine were previously mentioned to be used in treatment of various neurodegenerative diseases such as depression, sleep disorder, mood regulation, and AD. Etoperidone has been specifically reported to influence tau protein phosphorylation with the name Trazadone (its analogue) [31, 32]. Queuine is recently reported for preventing both tau phosphorylation and amyloid beta plague formation in AD [33]. However, to the best of our knowledge neither have been investigated for targeting AChE inhibition to cure AD, thus far. We further validated our results with *in vitro* cell culture experiments and compared with FDA approved control drugs. We investigated the cell viability effects of our candidate molecules on the SH-SY5Y neuroblastoma cell line using RTCA and MTT methods. We selected one synthetic (Donepezil) and one natural (Galantamine) FDA-approved AChE inhibitor as control molecules, so as to compare the effectiveness and safety of each candidate in the same region. RTCA and MTT analysis provided us to determine the safe (non-toxic) concentrations of each drug and predicted IC_50_ values set a relative comparison between the potencies of each drug as compared to controls. The promising results obtained for these molecules shed light on the development of the next step *in vivo* animal testing and offer hope for natural therapeutics that assist in the treatment of AD.

## Results and discussion

### *In silico* studies: Natural molecule library generation, virtual screening, molecular docking, molecular dynamics and ADMET

**Fig 1:**
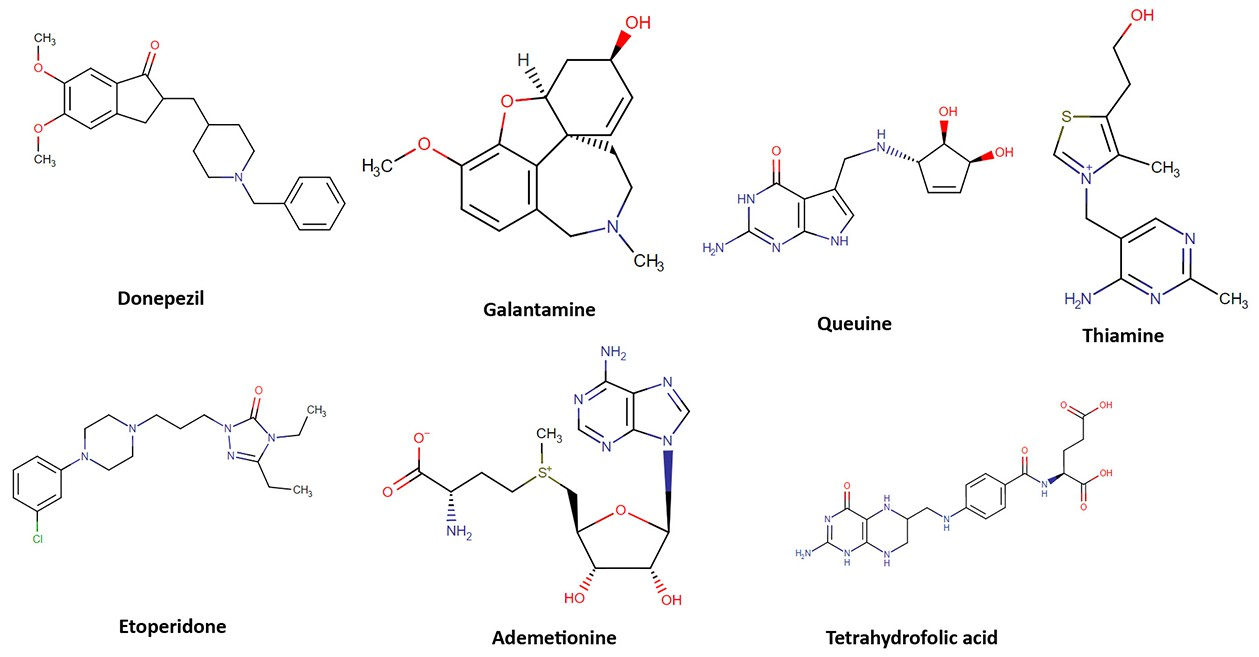
Chemical structures of Donepezil, Galantamine and five hit natural molecules investigated in this work as potential AChE inhibitors.

In the current study, we employed a systematic *in silico* approach to test the inhibitory activity of natural molecules (biogenics, metabolites and nutraceuticals) delivered from Drug Bank [34] and Zinc15 [35] databases against AChE. Our reference compounds were Donepezil (synthetic control) and Galantamine (natural control). Out of 121,587 natural molecules and conformers that are docked to AChE binding sites, 5 hit molecules were captured having high docking scores, performing essential interactions with CAS, PAS, Anionic and Acyl subsite sites and with acceptable ADMET properties. Fig 1 demonstrates the molecular structures of FDA approved control drugs (Donepezil and Galantamine) together with five leading molecules proposed as potential AChE inhibitors in the present study.

Properties of the above pictured natural hit molecules together with reference drugs are provided in Table 1. Queuine, Thiamine, Tetrahydrofolic acid and Ademetionine are classified as nutraceuticals; while Etoperidone is a metabolite. Thiamine, also known as vitamin B1, plays a key role in intracellular glucose metabolism and it is one of the most important vitamins for maintaining proper functioning of the heart, the nervous and the digestive systems of most living organisms [36]. Effect of thiamine in brain diseases has several ongoing clinical studies [37]. Ademetionine is a physiologic methyl radical donor involved in enzymatic transmethylation reactions and present in all living organisms. It possesses anti-inflammatory activity and has been used in treatment of chronic liver disease. S-Adenosylmethionine (SAMe) is used as a drug in Europe for the treatment of depression, liver disorders, fibromyalgia, and osteoarthritis. It has also been introduced into the United States market as a dietary supplement for the support of bone and joint health, as well as mood and emotional well being [38]. S-Adenosylmethionine has several completed clinical trials basically on neurodegenerative diseases such as bipolar, depression and AD [39]. S-Adenosylmethionine is metabolized to methionine by the help of cofactor folic acid, hence folate and methionine pathways are interlinked metabolic pathways. Folic acid is metabolized in the liver into tetrahydrofolic acid and folate [40, 41]. There are many studies based on folic acid and folate influence on AD [42, 43].

**Table 1:** Molecular properties of hit natural molecules compared to FDA approved AChE inhibitors.

Molecule name	DrugBank ID	Class	Target in AD treatment	Reference
**Queuine**	DB14732	nutraceutical	tau protein, amyloid-beta protein	[33]
**Etoperidone**	DB09194	metabolites	tau protein (Trazadone its analog)	[31, 32]
**Thiamine**	DB00152	nutraceutical	tau protein, amyloid-beta protein, AChE	[44, 45]
**Tetrahydrofolic acid**	DB00116	nutraceutical	tau protein, amyloid-beta protein	[40, 46]
**Ademetionine**	DB00118	nutraceutical	tau protein, amyloid-beta protein	[47, 48]
**Donepezil** ^a^	DB00843	FDA approved	AChE	[49, 50]
**Galantamine** ^b^	DB00674	FDA approved	AChE	[50, 51]

^a^synthetic control

^b^natural control

Queuine is a natural biochemical compound that can be found endogenously in the human body and plays an essential role in the generation of other critical bodily chemicals including tyrosine, serotonin, dopamine, epinephrine, norepinephrine, nitric oxide, lipids, and others [52]. The neuroprotective effect of Queuine is discovered very recently and there is a recent study based on its hindering effect of tau hyperphosphorylation and amyloid beta plague accumulation [33]. Etoperidone has a biphasic effect on the central transmission of serotonin. It presents the capacity to inhibit serotonin receptors but also to inhibit the reuptake of serotonin, norepinephrine and dopamine [53–55]. A few clinical trials exist for Etoperidone related drugs basically for treatment of sleep disorders and AD [56] and also several derivatives (trazodone, haloperidol) were reported to have an impact on AD [31, 32, 57]. Nevertheless, there are conflicting results related to the influence of Etoperidone related derivative molecules in aid of AD treatment. Hence, having quite limited number of studies targeting Queuine and Etoperidone and uncertainty about which cascades they are involved in, led us to put more effort into understanding their mechanism of action and relation in AD, if it exists.

**Fig 2:**
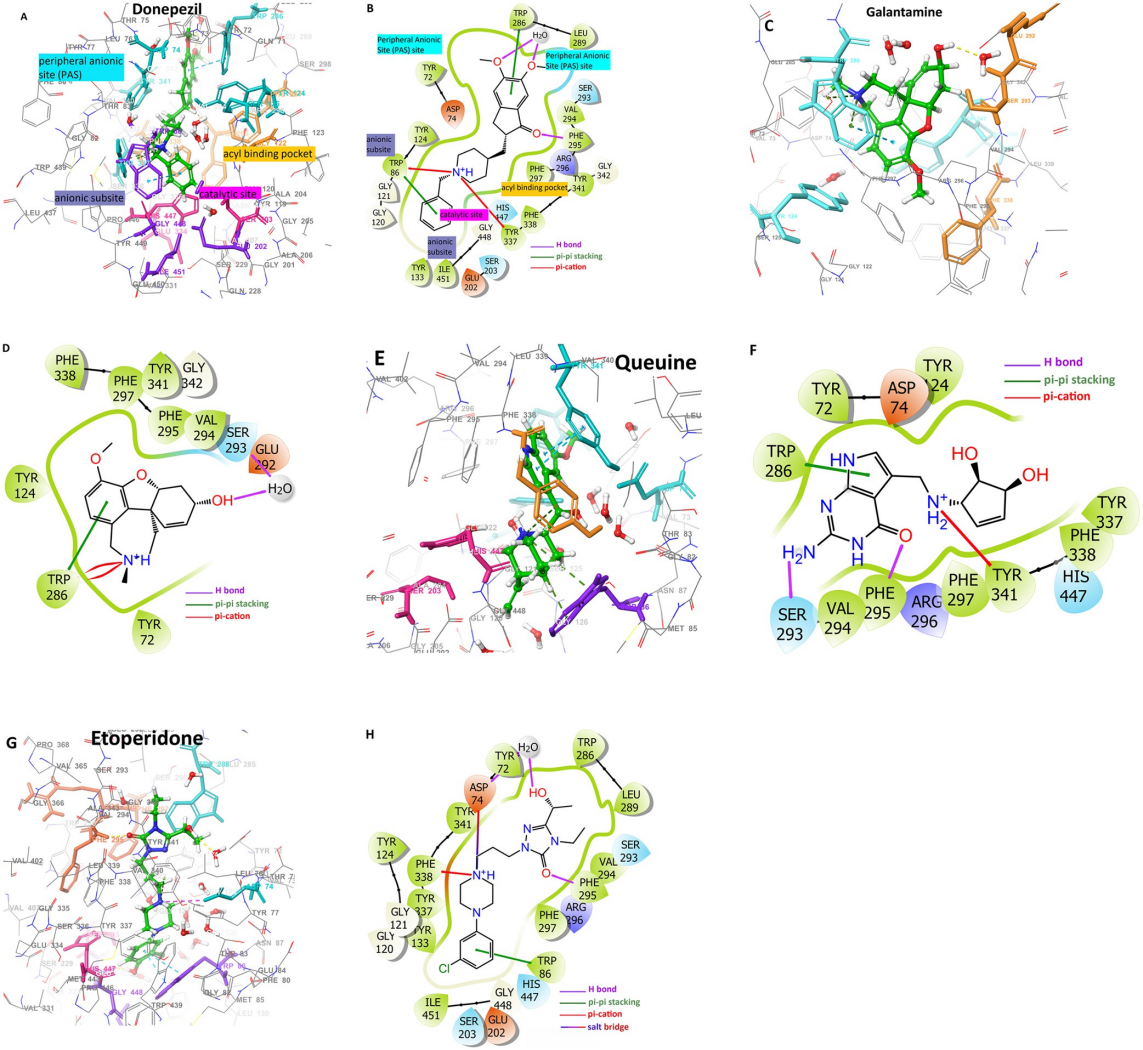
Docked conformations and important binding site interactions of suggested natural compounds as compared to control molecules. Panels A-B for Donepezil (3D-2D), C-D for Galantamine (3D-2D), E-F for Queuine (3D-2D) and G-H for Etoperidone (3D-2D) respectively. Coloring indicates: CAS (magenta), PAS site (cyan), anionic site (purple), and acyl binding site (orange).

Fig 2 depicts 3D and 2D docked conformations of reference molecules Donepezil and Galantamine together with Queuine and Etoperidone. PAS (cyan), CAS (magenta), Acyl (orange) and Anionic (purple) subsites having a crucial role in contributing to active site interactions in AChE cleft are also depicted (Fig 2A). Donepezil simultaneously binds to PAS and CAS sites (dual inhibitor) which makes it a potent inhibitor. Donepezil interacts with Asp 74, Tyr 124, Trp 286, Tyr 337, Tyr 341 in the PAS; Ser 203 and His 447 residues in the CAS subsites. PAS interactions involve π-π stacking and π-cation interactions; while polar interactions are observed with the CAS site (Fig 2B). Other than that, Donepezil also interacts with Anionic and Acyl binding pockets as well. Details of interacting residues and types of interactions are enlisted in Table 2 as well.

**Table 2:** Docking analysis of proposed natural compounds as compared with control molecules: Docking scores, interaction sites and residues involved in AChE binding site.

Ligand	AChE Docking Score(kcal/mol)	Interactions with AChE sites				Interacting residues	Interaction type
		CAS	PAS	Anionic	Acyl		
Queuine	-10.1		√		√	Tyr 341	π-cation
						Phe 295	H-bond
						Ser 293	H-bond
						Trp 286	π-π stacking
Etoperidone	-13.4	√	√	√	√	Tyr 72	H-bond
						Asp 74	H-bond
						Asp 74	salt bridge
						Tyr 341	π-cation
						Phe 295	H-bond
						Trp 86	π-π stacking
						Glu 202	H-bond
Donepezil	-14.8	√	√	√	√	Trp 86	π-π stacking
						Trp 86	π-cation
						Phe 338	π-cation
						Tyr 337	H-bond
						Phe 295	H-bond
						Trp 286	π-π stacking
Galantamine	-8.1		√		√	Trp 286	π-π stacking
						Trp 286	π-cation

Galantamine mainly interacts through PAS and Acyl binding pocket sites, with residues: Tyr 124, Trp 286, Tyr 337, Tyr 341, Phe 295, Phe 297, Phe 338 through π-π stacking and π-cation interactions (Fig 2C and 2D). Similar dual interactions are also obtained in the case of Etoperidone and Queuine which are shown to bind to PAS and CAS simultaneously performing many interactions with the corresponding sites. Queuine exhibits interactions with Asp 74, Tyr 124, Trp 286, Tyr 341 from PAS; Ser 293, Phe 295, Arg 296, Phe 297, and Phe 338 from Acyl binding pocket; His 447 from CAS. PAS site interactions are basically π-π stacking and π-cation type, Acyl pocket interactions are hydrophobic and polar type CAS interaction is accomplished (Fig 2E and 2F). Etoperidone resembles the interactions observed in Donepezil the most as compared to the other molecules. Similar to Donepezil, it interacts with residues from all four subsites. Belonging to the PAS site, residues like Asp 74, Tyr 124, Trp 286, Tyr 337 and Tyr 341 are involved in hydrogen bonding, π-cation and hydrophobic interactions (Fig 2G and 2H). For the interactions of Ademetionine, Thiamine and Tetrahydrofolic acid you may refer to S1A–S1F Fig.

For the CAS site, residues Ser 203 and His 447 are both involved as in the case of Donepezil, both performing polar interactions. As for the Anionic site, residues like Trp 86 and Glu 202 exhibit π-π stacking and hydrogen bonding. Finally, belonging to the Acyl binding pocket Phe 295 forms hydrogen bonding as well. In summary, we detected that Etoperidone performs similar interactions with AChE, in all four subsites as was also the case in Donepezil, while Queuine resembles Galantamine due to the fact that main interactions are concerned with PAS and Acyl binding pocket. Docking simulations provide us a relative comparison between the molecules and their interactions. However, what is more important than that is, the sustainability and stability of these interactions which would be elucidated by Molecular Dynamics simulations and MM-GBSA binding free energy calculations.

**Fig 3:**
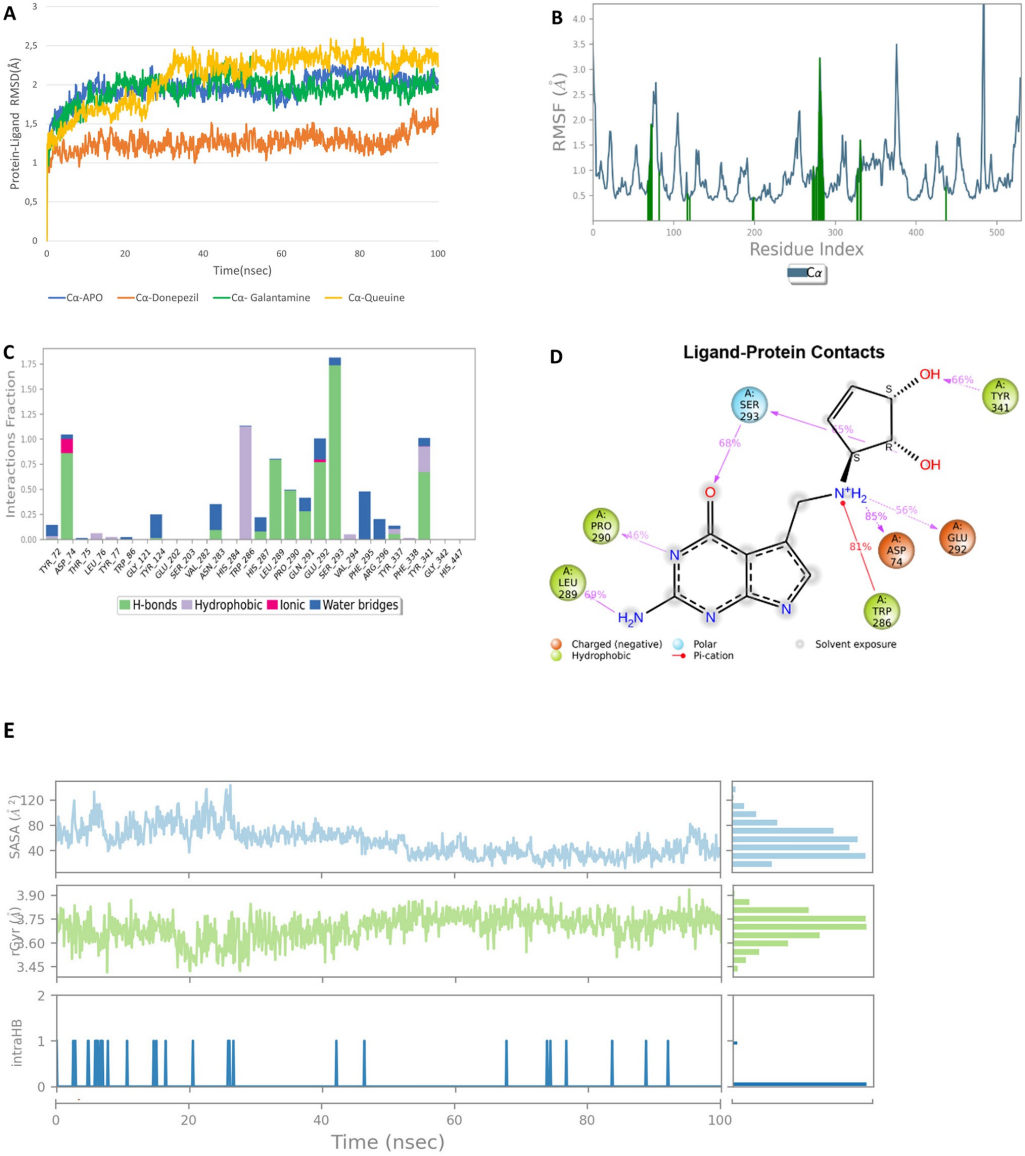
Molecular dynamics results of Queuine: RMSD (A), RMSF (B), protein-ligand fractions (C), protein-ligand contacts (% of simulation time) (D), SASA, radius of gyration and intramolecular hydrogen bonding (E) plots for Queuine.

We performed MD simulations for each molecule double runs for 100 ns. Fig 3 demonstrates the root mean square deviations (RMSD) (A), root mean square fluctuations (RMSF) (B), protein-ligand interactions (C-D), solvent accessible surface area (SASA), radius of gyration (rGyr) and intramolecular hydrogen bonds (intraHB) within the ligand (E), through MD simulations of Queuine-AChE complex system. RMSD results (Fig 3a) compare the Apo AChE with Donepezil, Galantamine and Queuine bound forms. All trends converges lower than 2.5 Å confirming the establishment of equilibration and a stable simulation. Donepezil RMSD values are lower than 1.5 Å, implying comparably a more stable complex formation; while Galantamine and Queuine exhibit similar profiles, i.e. similar protein-ligand stability, however, deviations being slightly higher than donepezil. Alterations brought to protein flexibility induced upon ligand binding can be clarified by plotting RMSF values (Fig 3B). Peaks indicate highly mobile (flexible) regions, while hinges point to more stable and less flexible regions of the protein during the simulation. The green vertical bars on the figure indicate the protein residues that interact with the ligand (Queuine). These residues are basically: Tyr 72, Asp 74, Tyr 124, Trp 286, Tyr 337, Tyr 341, Phe 338 from PAS site. Additionally, Phe 295, Arg 296 from Acyl binding site. Most significant interactions are through Glu 292, Ser 293 which both do not exist in Donepezil.

Protein interactions with Queuine are monitored throughout the simulation and categorized by type as provided in Fig 3C. The stacked bar charts are normalized over the course of trajectory. Values over 1.0 such as in the case of Asp 74, Trp 286, Glu 292 and Ser 293 are possible, meaning multiple contacts exist for the same protein residue. In Fig 3D, schematic of detailed ligand atom interactions that occur more than 30% of the simulation time with protein residues are pictured. Trp 286 and Asp 74 have significant interactions performed 81% and 85% of the simulation period in the case of Queuine.

Trp 286 makes π-cation interactions, while Asp 74 makes hydrogen bonding with tertiary amine nitrogen of Queuine. Following these Leu 289, Ser 293 and Tyr 341 also makes hydrogen bonding with Queuine more than 69%, 68% and 66% of the simulation time, respectively. Since there is not a notable interaction observed with CAS residues (His 447 or Ser 203) in Queuine, it seems that Queuine performs majority of its interactions with the PAS site residues, interactions being more stable than Donepezil (S2 Fig) and maintained all through the simulation time. Solvent accessible surface area (SASA), radius of gyration (rGyr) and intramolecular hydrogen bonding (intraHB) profiles are depicted on panel E. SASA values are recommended to be between 300–1000 Å2 [10]. Queuine exhibits acceptable range values being less than 120 Å2. Radius of gyration measures the extendedness of the ligand, being equivalent to its principal moment. rGyr values for Quine is observed to be between 3.4–3.9 Å. rGyr values were estimated to be between 5.2–5.5 Å in donepezil (S2 Fig panels A-D). Finally, intramolecular hydrogen bonding is depicted in panel E as well. Queuine shows significant intraHB interactions which actually may lead to a hindering in its interactions with protein. IntraHB was not observed in the case of Donepezil, Galantamine and Thiamine (S2–S4 Figs respectively).

**Fig 4:**
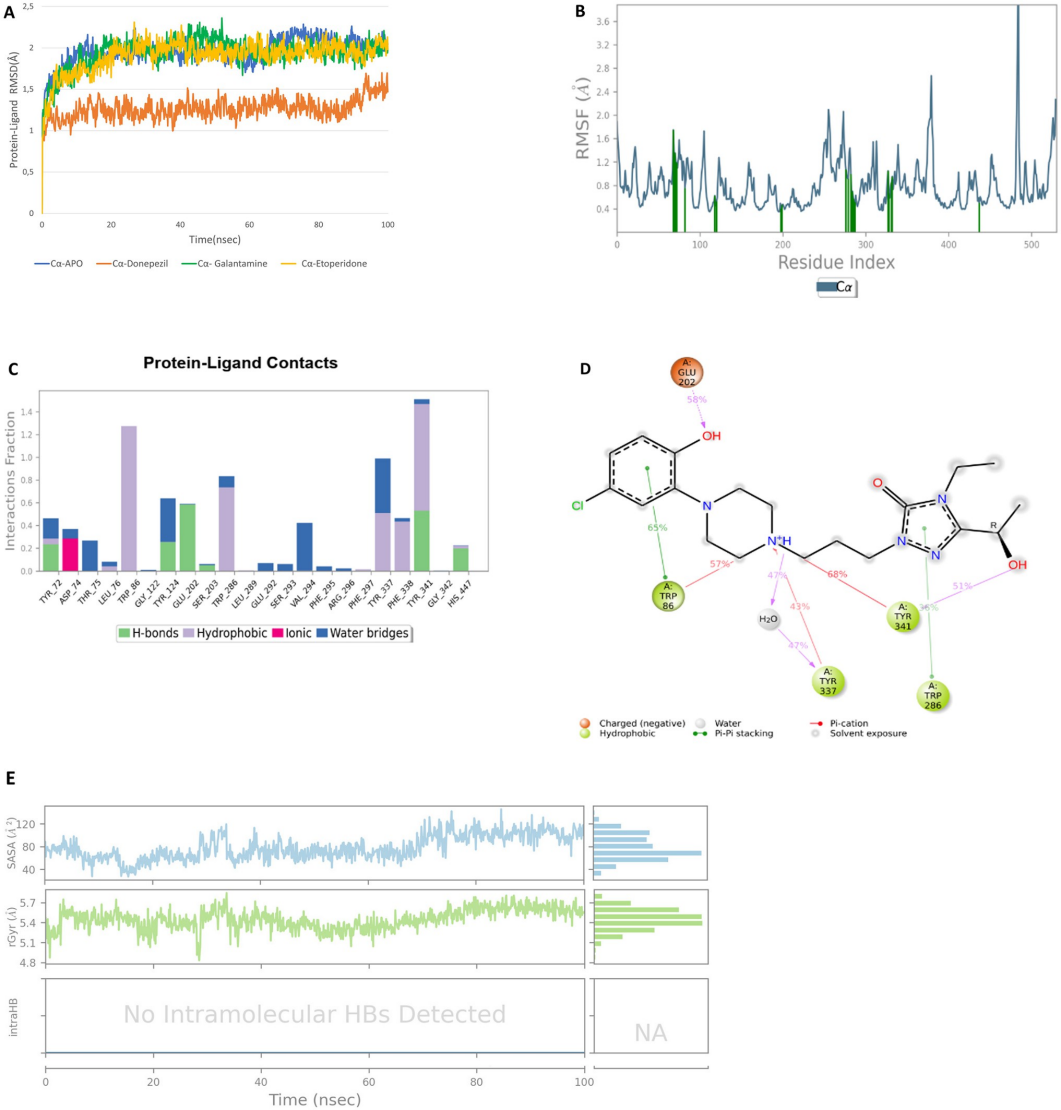
Molecular dynamics results of Etoperidone: RMSD (A), RMSF (B), protein-ligand fractions (C), protein-ligand contacts (% of simulation time) (D), SASA, radius of gyration and intramolecular hydrogen bonding (E) plots for Etoperidone.

Similar plots are provided for MD simulations of Etoperidone in Fig 4. RMSD of Etoperidone is lower than Queuine being less than 2.2 Å (Fig 4A), which is a closer value to that of Donepezil (1.5 Å) implying an equilibrated simulation and stable complex formation. RMSF plots (Fig 4B) really match with that of Queuine, major interactions performed with residues: Tyr 72, Asp 74,Tyr 124,Trp 286, Tyr 337 and Tyr 341 from PAS site; Trp 86 and Glu 202 from anionic site, Val 294 and Phe 338 from acyl binding site, Ser 203 and His 447 from catalytic site. Etoperidone interacts with all four functional domains of acetylcholine, similar or sometimes more pronounced as compared to Donepezil.

Protein-ligand interactions can be clarified in panels C and D. Bars indicating the interaction fractions through the simulation time for each residue (Fig 4C). Etoperidone interacts and maintains the interactions through essential residues. Specifically, catalytic site residues such as Ser 203 and His 447 exhibit a more prominent level. Ligand atom interactions that occur more than 30% of the simulation time with protein residues are demonstrated in Fig 4D. Residues like Glu 202, Trp 86, Trp 286, Tyr 337, Tyr341 pursue their interaction with Etoperidone almost more than 50% of the simulation time. Solvent accessible surface area (SASA), radius of gyration and intraHB profiles are depicted on Fig 4E. SASA values lie on the average between 40–80 Å2. Radius of gyration values are observed to be between 5.4–5.6 Å close to that of Donepezil. As in the case of Donepezil, no intramolecular hydrogen bonding has been detected in Etoperidone, which may have a favorable effect for sustainability of protein-ligand interactions.

**Fig 5:**
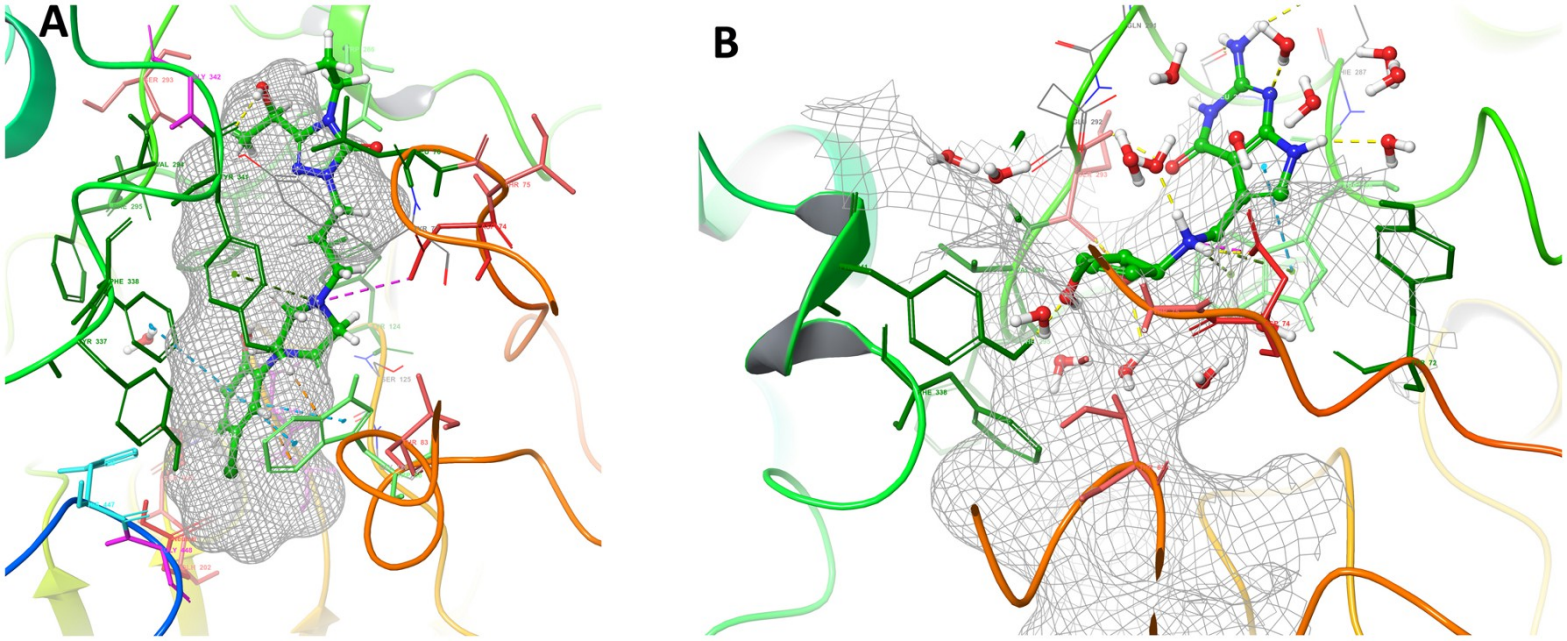
Final snapshots (100 ns) obtained from MD simulations: Etoperidone (A) and Queuine (B). Etoperidone stability is maintained within the binding cleft and interacts with CAS site (gray meshed surface); Queuine seems to diffuse out of the binding cleft and preserves its interactions with the PAS site.

Final snapshots obtained in the end of 100 ns Molecular Dynamics simulations are demonstrated in Fig 5. It is noteworthy to notice that Etoperidone is stable within the binding cleft and interact with CAS (gray dashed surface) till the end of the simulation, while Queuine seems to diffuse out and remain bound near the PAS. This would have another consequence associated with prevention of Aβ plague accumulation as previously pointed out in literature [58]. For the finalsnapshot figures of Donepezil, Galantamine and Thiamine, you may refer to S5A–S5C Fig, respectively).

Docking simulations and binding affinities may not realistically reflect actual binding energies, a more reliable comparison of free energy of ligand binding to protein can be performed by Molecular Mechanics-Generalized Born Surface Area (MM-GBSA) method [59]. MM-GBSA energies are calculated for each 100 ps frame and averaged values for 10 frames are provided in Table 3. The energy contributions from each component, namely, ΔEcoulomb, ΔEcovalent, ΔEsolvGB, ΔEvdW are tabulated. The total free energy of binding (ΔGbindtotal) values for Queuine, Etoperidone, Thiamine, Galantamine and Etoperidone are estimated as -71.9, -91.0, -68.2, -71.3, and -80.9 kcal/mol, respectively. The results are in agreement with docking, MD RMSF, MD protein-ligand interaction studies, which showed that Etoperidone is stable and maintained important contacts within the binding site more dominantly as compared to Queuine. Etoperidone has a higher binding energy as compared to both Galantamine and Donepezil, while Queuine has a comparable binding energy with Galantamine, but a lower value than Donepezil.

**Table 3:** MM-GBSA calculations from MD simulations.

Energy Terms	Queuine	Etoperidone	Thiamine	Galantamine	Donepezil
ΔE_coulomb_^a^	-107.43±11.02	-68.06±8.16	5.71±10.47	-80.85± 2.96	-66.44± 4.65
ΔE_covalent_^b^	4.52± 3.59	5.06±2.56	4.47±1.70	3.25± 2.22	3.58± 2.68
ΔE_solvGB_^c^	96.04± 5.12	72.92±5.27	-14.01±11.07	92.5± 2.95	91.40± 3.36
ΔE_vdW_^d^	-33.08± 4.66	-55.41±2.96	-35.87±4.16	-47.21± 1.92	-55.99± 3.83
**ΔG**_**bind**_ **total** ^e^	**-71.97 ± 10.79**	**-91.01 ± 8.76**	**-68.21**±6.35	**-71.28 ± 5.00**	**-80.91± 8.69**

^a^ Coulomb energy

^b^ Covalent binding energy

^c^ Generalized Born electrostatic solvation energy

^d^ Van der Waals energy

^e^ Total free energy of binding

As far as the druglike properties are concerned, all potential hit molecules follow Lipinski’s rule of five [60, 61] without any violation as tabulated in Table 4. In addition, the blood-brain barrier permeability (BBB) values are all positive, implying possible penetration through the brain barrier. Molecules showed no toxicity in mutagenic potential (AMES) tests.

**Table 4:** Predicted druglike and ADMET properties of hit natural AChE inhibitor molecules compared to donepezil and galantamine.

Molecule name	MW^a^ (g/mol)	log P^b^	HBD^c^	HBA^d^	TPSA^e^ (Å)	BBB^f^	HIA^g^ (%)	AMES^h^ toxicity
Queunine	277.3	-1.6	6	5	140	+	94.6	-
Etoperidone	377.9	2.8	0	3	46.3	+	100	-
Thiamine	265.4	-2.1	2	3	104.2	+	90	-
Donepezil	379.5	4.1	0	4	38.7	+	98.4	-
Galantamine	287.4	1.2	1	4	41.9	+	99.5	-

^a^ Molecular Weight

^b^ octanol/water partition coefficient from ALOGPS [62]

^c^ Hydrogen bond donor from SwissADME [63]

^d^ Hydrogen bond acceptor from SwissADME

^e^ Topological polar surface area from SwissADME

^f^ Blood-brain barrier permeability from admetSAR [64]

^g^ Human intestinal absorption from admetSAR

^h^ Mutagenic potential from admetSAR

### *In vitro* biological evaluation: Effects of the reagents on cell viability

The effects of donepezil, galantamine, queuine, etoperidone, and thiamine on cell viability proliferation were determined in the human neuroblastoma cell line SH-SY5Y by xCELLigence Real Time Cell Analysis (RTCA) system and 3-(4,5-Dimethylthiazol-2-yl)-2,5-Diphenyltetrazolium Bromide (MTT) assays. For this, SH-SY5Y cells were exposed to indicated concentrations of Donepezil (5, 10, 15, 25 and 50 μM), Galantamine (5, 10,16, 25, 50 and 100 μM), Queuine (0,01, 0,05, 0,25, 1,25 and 5 μM), Etoperidone (25, 50, 60, 70, 75, 80, and 100 μM) and Thiamine (150, 300, 600, 1200, 2400 and 4800 μM) for 48 h, and relative cell viability was calculated.

**Fig 6:**
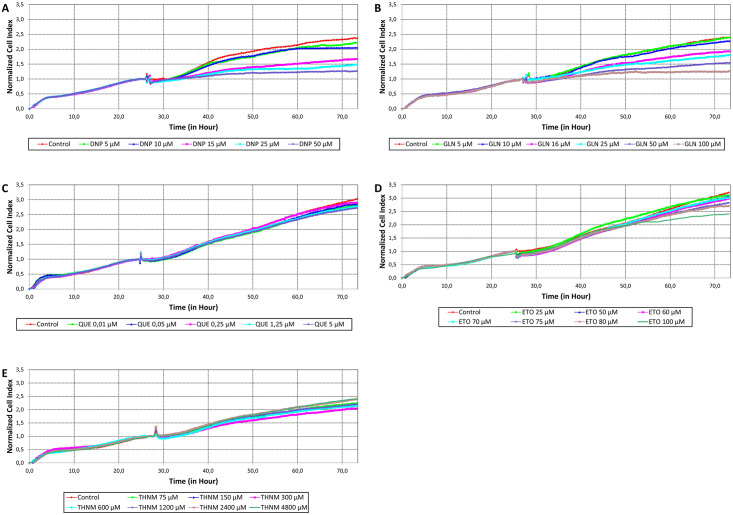
The results of the RTCA analysis: The cells were not treated with any drug for the first 24 h. After 24 hour, different concentrations of donepezil (A), galantamine (B), queuine (C), etoperidone (D), and thiamine (E) were applied. Following drug application, cell index was measured over time for 48 hours and plotted.

The cell index graphs obtained from the xCELLingence RTCA system were demonstrated in Fig 6, panels A to E for Donepezil, Galantamine, Queuine, Etoperidone and Thiamine, respectively. The cell index percentage graphs obtained from the xCELLingence RTCA are presented in S6A–S6E Fig. According to the cell index values, no cytotoxicity was observed at concentrations up to 15 μM for Donepezil, 10 μM for Galantamine, 1.25μM for Queuine, 70 μM for Etoperidone, and 4800 μM for Thiamine 5μM, 10μM, and 15μM for donepezil, 5μM and 10μM for galantamine, 0.25μM for queuine, 50μM, 60μM, and 70μM for etoperidone, and 2400μM for thiamine for 48 hours, and no significant cytotoxicity was observed. Thiamine, on the other hand, showed no significant cytotoxicity at any applied concentration. However, after 48 hours, it was observed that the survival rate of cells treated with 100μM Galantamine was significantly lower than that of the control group (p < 0.001). In addition, it was found that 25μM donepezil, 25–50μM galantamine, 5 μM Queuine and 100μM etoperidone significantly reduced cell viability compared to the control group (p < 0.001). (and higher concentrations for each reagent) showed significant cytotoxic effects on cell viability (p < 0.001).

All concentrations used in RTCA were tested on SHSY5Y cells using the MTT assay to confirm the effect of reagents on cell viability. According to the MTT assay findings, similar to the RTCA results, Donapezil, Galantamine, Etoperidone and Queuine induced a cytotoxicity in cells at increasing concentrations in a concenration dependent manner. While cell viability was close to the control treated with 15 μM for Donepezil, 10 μM for Galantamine, 1.25μM for Queuine, and 70 μM for Etoperidone, and below, indicating no cytotoxicity for these concentrations, Thiamine did not show any cytotoxic effect at any concentration, as in the RTCA results. On the other hand, again in correlation with RTCA findings, 15 μM for Donapezil, 25 μM for Galantamine, 5 μM for Queuine and 100 μM for Etoperidone.

**Fig 7:**
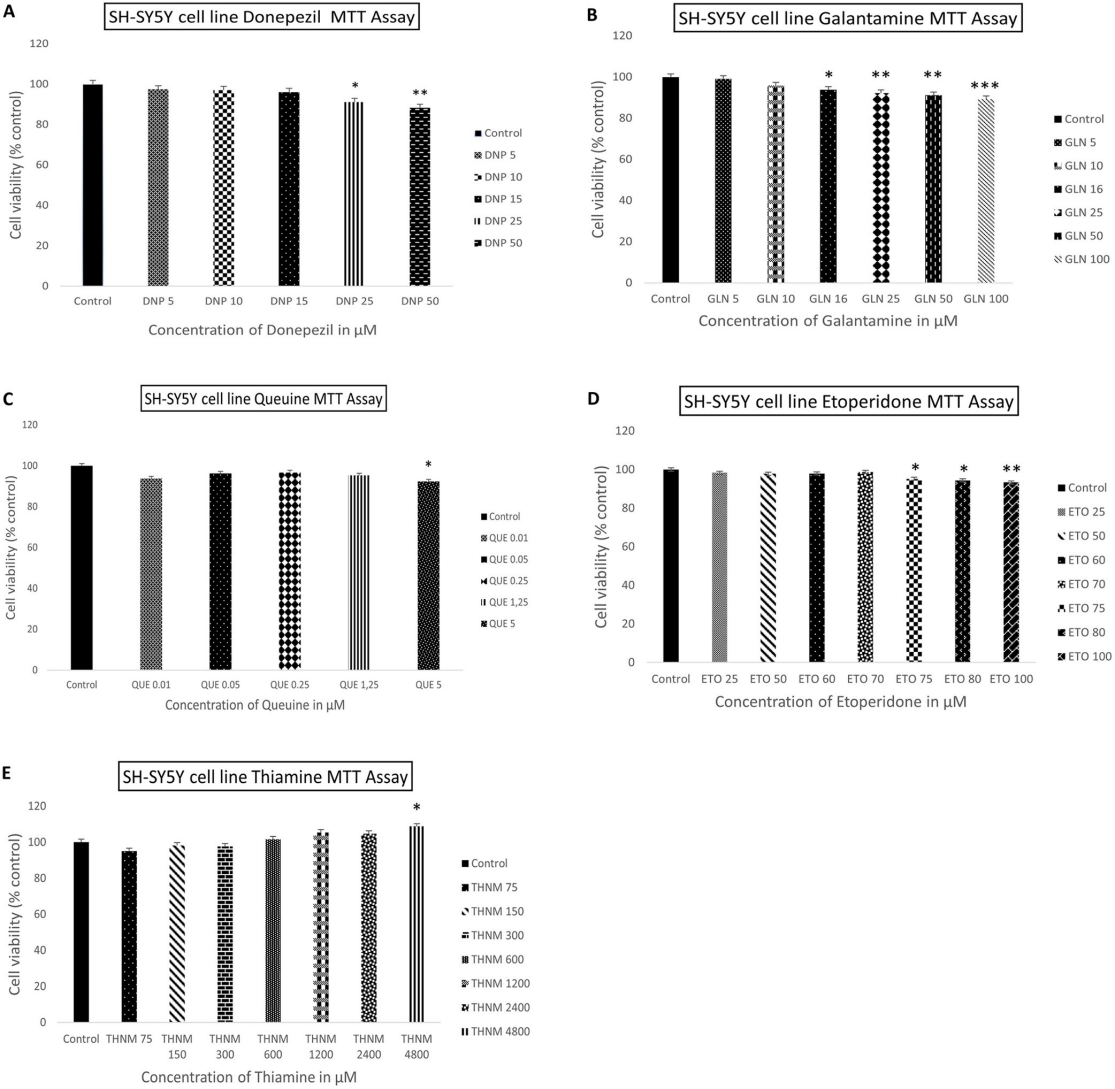
Percentage graph of cell viability of SH-SY5Y cells compared to the control group after exposure to different concentrations: Donepezil (A), Galantamine (B), Queuine (C), Etoperidone (D), and Thiamine (E) for 48 hours. The results represent the average ± SEM of the results from each experiment, which were repeated three times at different times with the same concentration ranges. Compared to the control, *P\0.05, **P\0.01, ***P\0.001.

The effects of Donepezil, Galantamine, Queuine, Etoperidone, and Thiamine on cell density were also examined in the SH-SY5Y neuroblastoma cell line using the MTT assay. The cell index percentage graphs obtained from the RTCA device (S6A–S6E Fig) were in agreement with those obtained from the MTT results (Fig 7).

According to our RTCA and MTT results, the applied candidate molecules had a very similar effect on the viability of the cells at the same concentrations, confirming each other. xCELLigence RTCA system use impedance-based technology to monitor live cell proliferation, morphological change, and attachment quality continually and in real-time. In general, an increase in the cell index value is seen in accordance with the number of proliferating cells that attach to the culture substrate in a healthy manner. But occasionally, the substances that are applied to the cell may cause cell enlargement by altering their morphology as a result of stress or other factors. When this occurs, the cells may show more adhesion with the culture surface, and even if the cells are unhealthy and do not proliferate, the cell index value may increase, giving a false positive value. As a result, the MTT assay’s accuracy in measuring cell viability is combined in this study with the xCELLigence system’s capability to track cell proliferation by attachment quality in real time.

In this study, the safe optimal concentrations of the supplied molecules *in vitro* were established based on the findings of both RTCA and MTT tests. Accordingly, the safe optimum concentrations that do not give harm cell viability are as follows, respectively; 15 μM for Donepezil, 10 μM for Galantamine, 1.25μM for Queuine, and 70 μM for Etoperidone. Considering that no dose of Thiamine applied exhibited harmful effects, biological efficacy research will continue when more than one dose is tried in biological activity tests.

As a result, the optimal doses identified here will be used in future research to carry out the *in vitro* and *in vivo* inhibitory activity of the molecules whose *in silico* activity was determined in this work against the acetylcholinesterase enzyme. The RTCA results showed that our candidate molecules did not have a negative effect on cell growth or viability on the cell line. In summary, the MTT and RTCA results confirmed that our candidate molecules were effective in passing through the cell barrier and exerting an effect on the cell line. *In vitro* validation of computational results is conducted through RTCA and MTT. The resulting half inhibitory concentration values (IC_50_) are presented in Table 5.

**Table 5:** IC_50_ values for selected test compounds as AChE inhibitors.

Test Compound	IC_50_ ± SEM (μM)
Donepezil	222,23 ± 4,06
Galantamine	556,01 ± 3,55
Thiamine	18780,34 ± 4,91
Queuine	70,90 ± 1,81
Etoperidone	712,80 ± 2,19

## Materials and methods

### Protein preparation

The crystal structures of acetylcholinesterase (AChE, pdb id: 4EY7) complexed with small molecule inhibitors were obtained from the Protein Data Bank and prepared using Schrödinger’s Maestro Molecular Modeling Suite [65, 66] and protein preparation wizard module [67]. Protein structure retrieved is first corrected for bond orders and missing hydrogen atoms. All heteroatoms other than the native ligand are removed. But the water atoms within 5 Å around the binding cleft were kept. In case there are any missing side chains or missing loops, Prime module was used to fill in these gaps (though this structure did not have any). Protonation states were generated using PROPKA at pH: 7.0. Finally, restrained minimization was carried out using 0.3 Å RMSD and OPLS2005 (Optimized potentials for liquid simulations 2005) force field [68].

### Ligand preparation

Prior to all docking simulations, the ligands were prepared using the LigPrep module of Maestro, Schrödinger software [66, 67]. Ionization states and tautomers were generated using Epik at pH: 7.0 ± 2.0 [69]. Stereoisomers were generated using chiralities from the 3D structure of the ligands. Natural source molecules (metabolites, nutraceuticals, biogenic) delivered from DrugBank [34] and Zinc [35] databases comprise of 2,674 and 83,830 molecules respectively. Totally 86,504 molecules were collected and a natural product library was prepared. Applying Lipinski’s rule of five [60, 61], the library was pre-filtered resulting in 85,716 molecules that are prepared by LigPrep, generating 121,587 conformers that will be directly docked into AChE binding site.

### Molecular docking

Molecular docking calculations were conducted using the Glide SP (standard precision) algorithm [70] in the Ligand Docking Module of Schrödinger Suite. The grid box was generated around the AChE binding cleft centered on the centroid of the co-crystal ligand using the Receptor Grid Generation module. Size of the grid box was selected to enable docking of the ligands with length ≤ 10 Å. Same grid file was used in all docking simulations for a reliable comparison. All the docked ligands were prepared by the LigPrep module prior to docking as explained above. Ligands were kept flexible and Epik state penalties were added to docking scores. To validate the docking protocol, co-crytallized ligand was redocked and the RMSD between co-crystal and docked donepezil conformation was calculated to be 1.9 Å. 85,716 natural source molecules prepared by LigPrep, generating 121,587 conformers were docked into acetylcholinesterase binding site. Molecules having high binding affinity (lower than -10 kcal/mol) were filtered.

### Molecular dynamics

Molecular dynamics simulations were performed with selected hits for 100 ns for docked complex systems using Desmond [71]. A total of 1 μs simulations were performed including AChE apo form, also for systems including queuine, etoperidone, thiamine, donepezil (control) and galantamine (control) docked in AChE. For each system, runs were performed twice. The size of the system box was 10 × 10 × 10 Å, and the box shape was orthorhombic. Solvent model was TIP3P and 0.15 M NaCl salt was added to the system for neutralization by excluding ions and salt within 20 Å of the ligand. In all MD simulations, the temperature was fixed to 300 K with NPT ensemble Nose–Hoover chain termostat [72], and the pressure was fixed to 1.0 bar Martyna–Tobias–Klein barostat [73]. All systems were prepared for MD simulations using OPLS2005 force field and RESPA integrator [74]. Interactions for coulomb, non-bonded, van der waals were calculated by OPLS2005. 100 ps time step was used. Trajectory analyses were performed with recorded 1,000 trajectory frames from each simulation throughout the MD simulations.

### Molecular mechanics, the generalized born model and solvent accessibility (MM-GBSA) calculations

**Fig 8:**
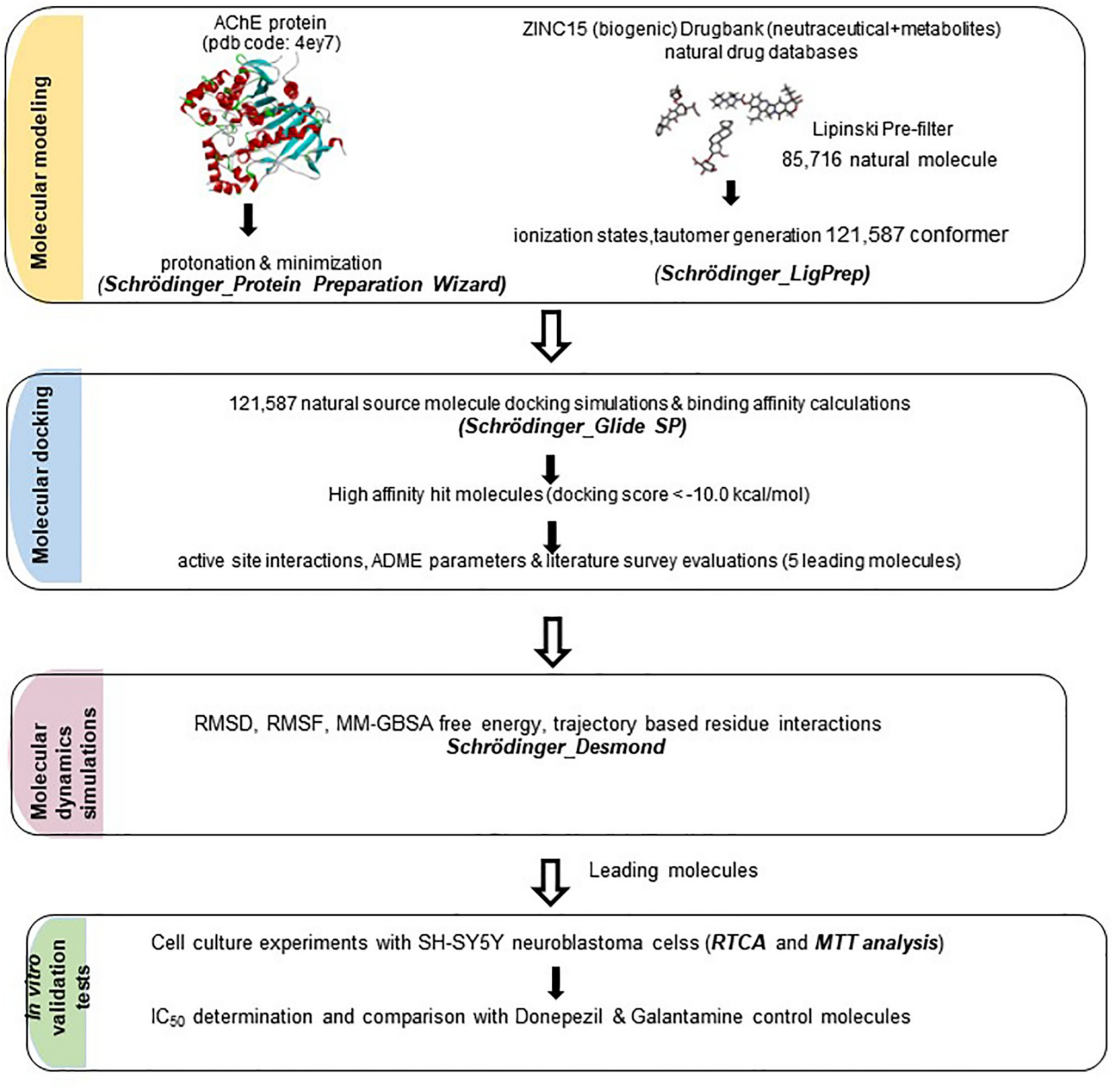
Schematic representation of the workflow.

Docking scores may only give a relative comparison between the compounds. A more thorough analysis on free energy of binding is carried out by the MM-GBSA method. To perform more accurate binding free energy calculation, Prime MM-GBSA module of Schrödinger Suite was used. The binding free energies of the protein–ligand complexes of queuine, etoperidone, thiamine, and control molecules Donepezil and Galantamine were calculated. Ligand and receptor files were created for 1001 frames of 0.1 ns from the molecular dynamics simulation file recorded for 100 ns for all compounds. MM-GBSA calculations were performed for 100 frame intervals and average values of 10 frames were taken for the free energy calculations. VSGB 2.0 model, which is reported to be suitable for biological function modeling and structural drug discovery, was used as the solution model [75]. Residues 3 Å from the ligand were kept flexible. The minimization as sampling method and OPLS2005 as force field were used, respectively. The schematic of the workflow is depicted in Fig 8.

### Drug likeness and ADMET analysis

Identified hit molecules and control molecules are subjected to drug-likeness by checking Lipinski’s rule of five [60, 61] violation. To predict ADMET (absorption, distribution, metabolism, excretion and toxicity) SwissADME [63] and admetSAR [64] servers were used.

### *In vitro* studies

The chemical compounds used in the experiments were purchased from the following suppliers: Donepezil hydrochloride (Cat. No: D6821) and Galantamine hydrobromide (Cat. No: G1660), Thiamine hydrochloride (Cat No: T1270) were obtained from Sigma-Aldrich. Etoperidone hydrochloride (Cat. No: sc-211494) and Queuine hydrochloride (Cat No: sc-394021) were purchased from Santa Cruz Biotechnology.

### Cell culture

This study utilized SH-SY5Y (human neuroblastoma) cell lines. The SH-SY5Y cell line (CRL-2266) was provided by ATCC. After dissolving the cell line stored in a nitrogen tank for long-term use, the cells were removed from the culture dish with a 0.25 Trypsin/EDTA solution when they reached 70% density in the culture flasks. The cells were incubated in DMEM (Capricorn) supplemented with 10% heat-inactivated fetal bovine serum (FBS) and 0.1 mg/ml of penicillin at 37°C with 5% CO_2_. The medium was refreshed every 48 hours, and passages were made every 5 days based on cell confluency.

### Preperation of drugs

Donepezil Hydrochloride (obtained from Sigma Aldrich, St. Louis, MO, USA), Galantamine Hydrobromide (obtained from Sigma Aldrich, St. Louis, MO, USA), Queuine Hydrochloride (obtained from Santa-Cruz) and Etoperidone Hydrochloride (obtained from Santa-Cruz) were all dissolved in dimethyl sulfoxide (DMSO). Thiamine Hydrochloride (obtained from Sigma Aldrich, St. Louis, MO, USA) was dissolved in double distilled water (ddH_2_O) Galantamine we purchased was extracted from Lycoris sp. plant.

### RTCA of candidate drugs in SH-SY5Y cell line

The optimum safe concentrations of five drugs that will be used in the cell culture, donepezil, galantamine, queuine, etoperidone and thiamine, were determined using a real-time cell analysis (RTCA, xCELLigence) and MTT cell viability assay. RTCA system is an analytical technique that follows cellular events such as proliferation by measuring the electric impedance passing through microelectrodes integrated beneath 96-well special plates in real-time. A 16-well e-plate compatible with the RTCA device was used, with 8 wells by 2. Firstly, 100 μl of cell culture was added to the plates to obtain the background density. Then, 3x104 cells were seeded in 100 μl of culture medium on top of the culture. After 24 h, the determined concentration ranges were applied.

For donepezil, studies have shown that there was no effect on cell viability up to 10 μM and a decrease in cell viability after 15 μM [76]. Therefore, in this study, the concentrations of 5-10-15-25-50 μM were applied to find the optimum dose. For galantamine, it has been shown that there was no significant decrease in cell viability up to 10 and 16 μM [77]. This study was taken as a reference, and the concentrations of 5-10-16-25-50-100 μM were applied for galantamine. There was only one study on a different cell line for quinine [41]. The concentration range of 0.01–0.05–0.25–1.25–5 μM was chosen for SH-SY5Y cell line based on the study. The concentration range of 25-50-60-70-75-80-100 μM was chosen for etoperidone based on cell viability tests conducted with its analogue, trazadone [78]. The concentration range of 75-150-300-600-1200-2400-4800 μM was selected for thiamine [79].

For each selected dose, the drug was applied to two different wells. The first two wells were designated as the control group without drug application. The growth curve data was obtained from the graph generated by the device 48 h after drug application. The RTCA was repeated three times for each drug group. The IC_50_ calculations and the percent cell viability graphs compared to the control group were obtained from the data obtained from the three repeats.

### Cell viability analysis of candidate drugs in SH-SY5Y cell line using MTT assay

The MTT (3-(4,5-dimethylthiazol-2-yl)-2,5-diphenyltetrazolium bromide) assay is a commonly used colorimetric assay to measure cellular viability. The test is based on the principle that live cells are able to reduce yellow MTT compound to a blue formazan product. The amount of formazan produced can be measured by determining the absorbance of the solution at a specific wavelength, which is proportional to the number of viable cells. This assay is widely used in cell biology and drug discovery research to evaluate the cytotoxic effects of various agents on cell viability.

In this study, the MTT assay was selected as the cell viability test. 12,500 cells were seeded per well in a 96-well plate. The cells were then incubated at 37°C with 5% CO2 for 24 h. After 24 h, the old medium was removed and drugs prepared by serial dilution in 100 μl of DMEM within the determined concentration range were applied to three wells, with one well selected as a control (untreated). The plate was then incubated at 37°C with 5% CO2 for another 48 h. After 48 h, 110 μl of 10% MTT (Roche) solution in DMEM was added to each well, and two empty wells were added with only MTT solution for background measurement. After 4 h, 100 μl of solubilizing buffer was added to each well. The plate was incubated at 37°C with 5% CO2 for 16 h. After 16 h, the cell number in each sample was determined by measuring the absorbance spectrophotometrically at 480 nm with a 570 nm background using a microplate reader (OMEGA). The data was used to determine the percentage relative to the control and create a dose-cell viability graph.

### Statistical analysis

All the analysis data from the RTCA device and the data generated from the MARS application using the microplate reader (OMEGA) were calculated in the GraphPad Prism 9.5.1 version using the ordinary one-way ANOVA multiple comparisons Tukey test. The results were graphically represented in Excel. Logarithmic calculations were made from the dose-response graph in Excel to obtain the IC_50_ values.

## Conclusions

This work aims to focus on new natural source compounds that may compete with FDA approved drugs like Donepezil, by better activity, much less toxicity and a possible multi-target effect. We carried out *in silico* discovery validated by *in vitro* optimum safe concentrations to propose Queuine and Etoperidone as natural compounds to be evaluated as potent AChE inhibitors. Queuine and Etoperidone both bind to the catalytic cleft with high affinity and lower binding free energy values as compared to Donepezil and Galantamine which are utilized for synthetic and natural controls. Etoperidone has been observed to be more stable as compared to Queuine within the binding cleft. Queuine was observed to diffuse to the PAS site and remain bound there. PAS site has been previously reported to have an influence on the amyloid cascade hypothesis. As a future work, the effect of Queuine may be better clarified by investigating amyloid beta plague accumulations in its presence and absence in both *in vitro* and *in vivo* AD models. We obtained promising inhibition values for: Queuine (IC_50_ = 70,90 μM), Etoperidone (IC_50_ = 712,80 μM), Thiamine (IC_50_ = 18780,34 μM) which may be compared to that of Galantamine (IC_50_ = 556,01 μM) and Donepezil (IC_50_ = 222,23 μM). Much lower IC_50_ value of Queuine indicates its significant effect even at very low concentrations. Thiamine has quite high IC_50_ value as compared to all the others which means that it is non-toxic and safe even at elevated concentrations. These compounds possess tertiary amine groups and good drug-like properties, including BBB permeability. Specifically we propose that Etoperidone is possibly involved in the cholinergic hypothesis, while Queuine may have an impact not only on cholinergic but also on other AD related cascades such as inhibition of Aβ plague accumulation as well. Queuine may act as a multi-target therapeutic, that would be further tested and clarified by future work.

## Supporting information

S1 Fig Docked conformations and important binding site interactions of suggested natural compounds as compared to control molecules. Panels A-B for Thiamine (3D-2D), C-D for Ademetionine (3D-2D), E-F for Tetrahydrofolic acid (3D-2D). Coloring indicates: CAS (magenta), PAS site (cyan), anionic site (purple), and acyl binding site (orange).
(TIF)

S2 Fig Molecular dynamics results of Donepezil: RMSF (A), protein-ligand fractions (B), protein-ligand contacts (% of simulation time) (C), SASA, radius of gyration and intramolecular hydrogen bonding (D) plots for Donepezil. (TIF)

S3 Fig Molecular dynamics results of Galantamine: RMSF (A), protein-ligand fractions (B), protein-ligand contacts (% of simulation time) (C), SASA, radius of gyration and intramolecular hydrogen bonding (D) plots for Galantamine. (TIF)

S4 Fig Molecular dynamics results of thiamine: RMSD (A), RMSF (B), protein-ligand fractions (C), protein-ligand contacts (% of simulation time) (D), SASA, radius of gyration and intramolecular hydrogen bonding (E) plots for Thiamine. (TIF)

S5 Fig Final snapshots (100 ns) obtained from MD simulations: Donepezil (A), Galanthamine (B) and Thiamine (C). All three molecules maintain their stability within the binding pocket throughout the simulation trajectory.
(TIF)

S6 Fig Percentage graph of cell index of SH-SY5Y cells compared to the control group after exposure to different concentrations: Donepezil (A), Galantamine (B), Queuine (C), Etoperidone (D), and Thiamine (E) for 48 hours. The results represent the average ± SEM of the results from each experiment, which were repeated three times at different times with the same concentration ranges. Compared to the control, *P\0.05, **P\0.01, ***P\0.001.
(TIF)
