# Supplementary figures and images for: Proposing novel natural compounds against Alzheimer’s disease targeting acetylcholinesterase
Source: PLoS One. 2023 Apr 27;18(4):e0284994. doi: 10.1371/journal.pone.0284994 (PMC10138233; doi:10.1371/journal.pone.0284994)

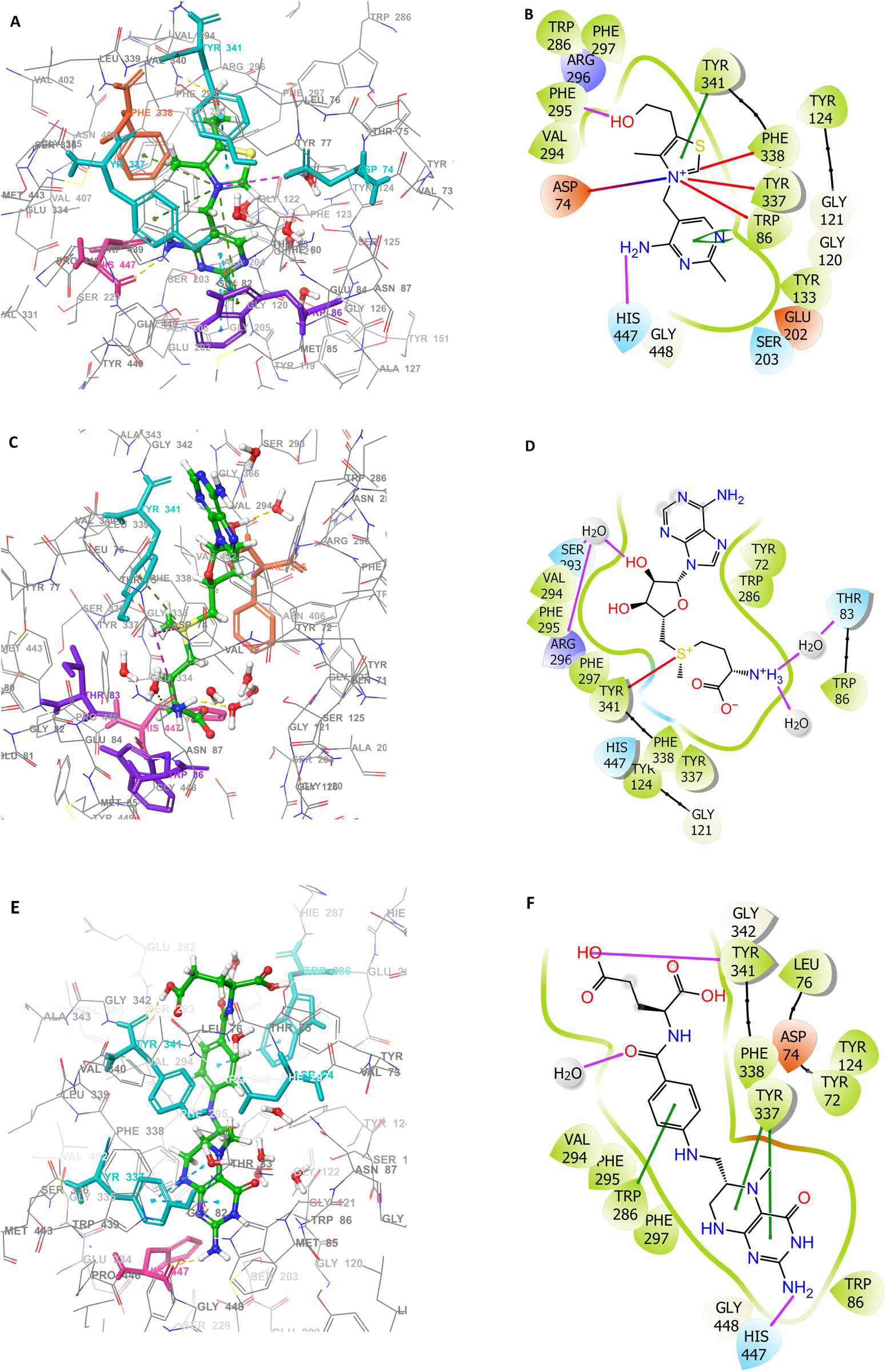

Supplement: S1 Fig — Panels A-B for Thiamine (3D-2D), C-D for Ademetionine (3D-2D), E-F for Tetrahydrofolic acid (3D-2D). Coloring indicates: CAS (magenta), PAS site (cyan), anionic site (purple), and acyl binding site (orange). (TIF) [file pone.0284994.s001.tif]

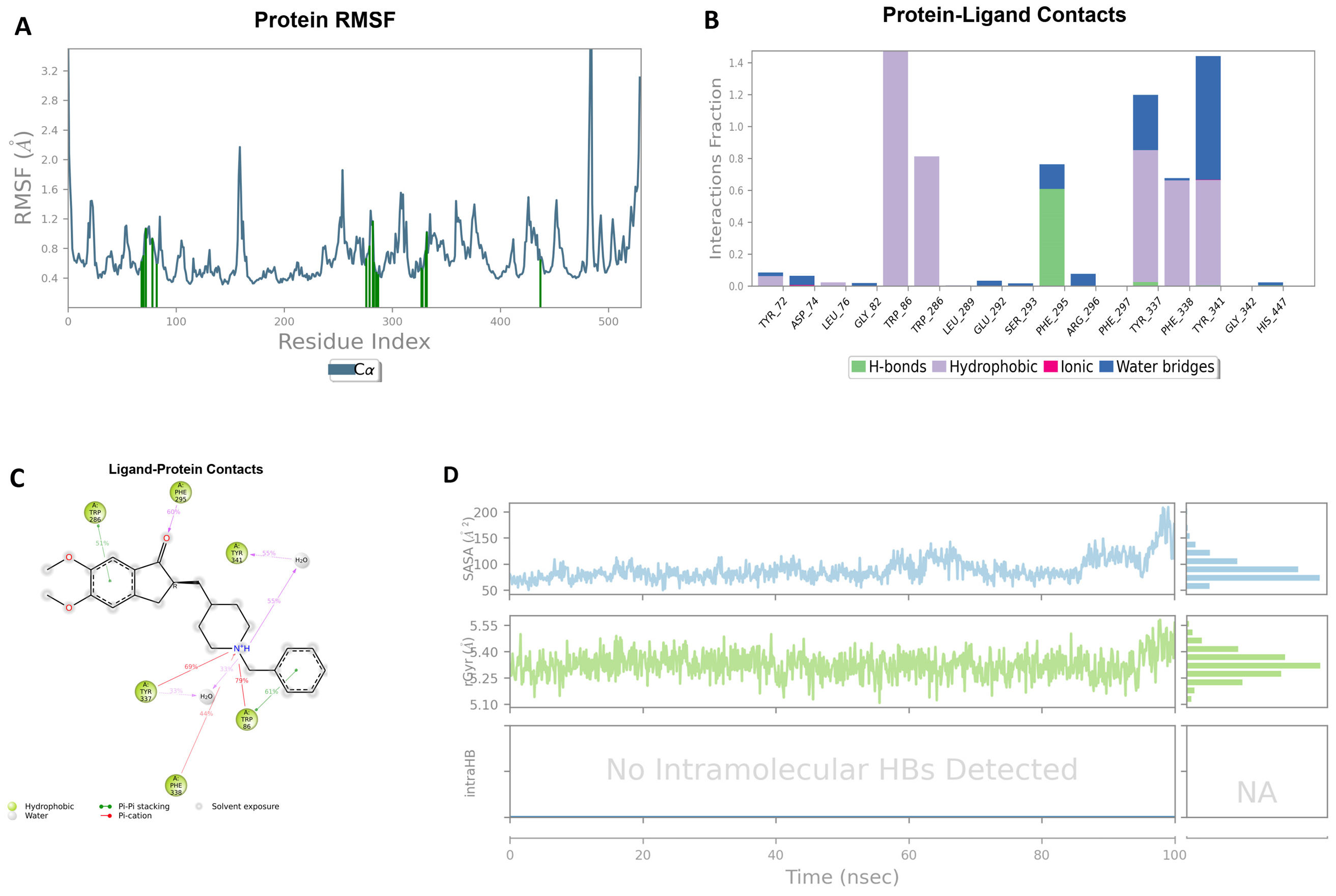

Supplement: S2 Fig — (TIF) [file pone.0284994.s002.tif]

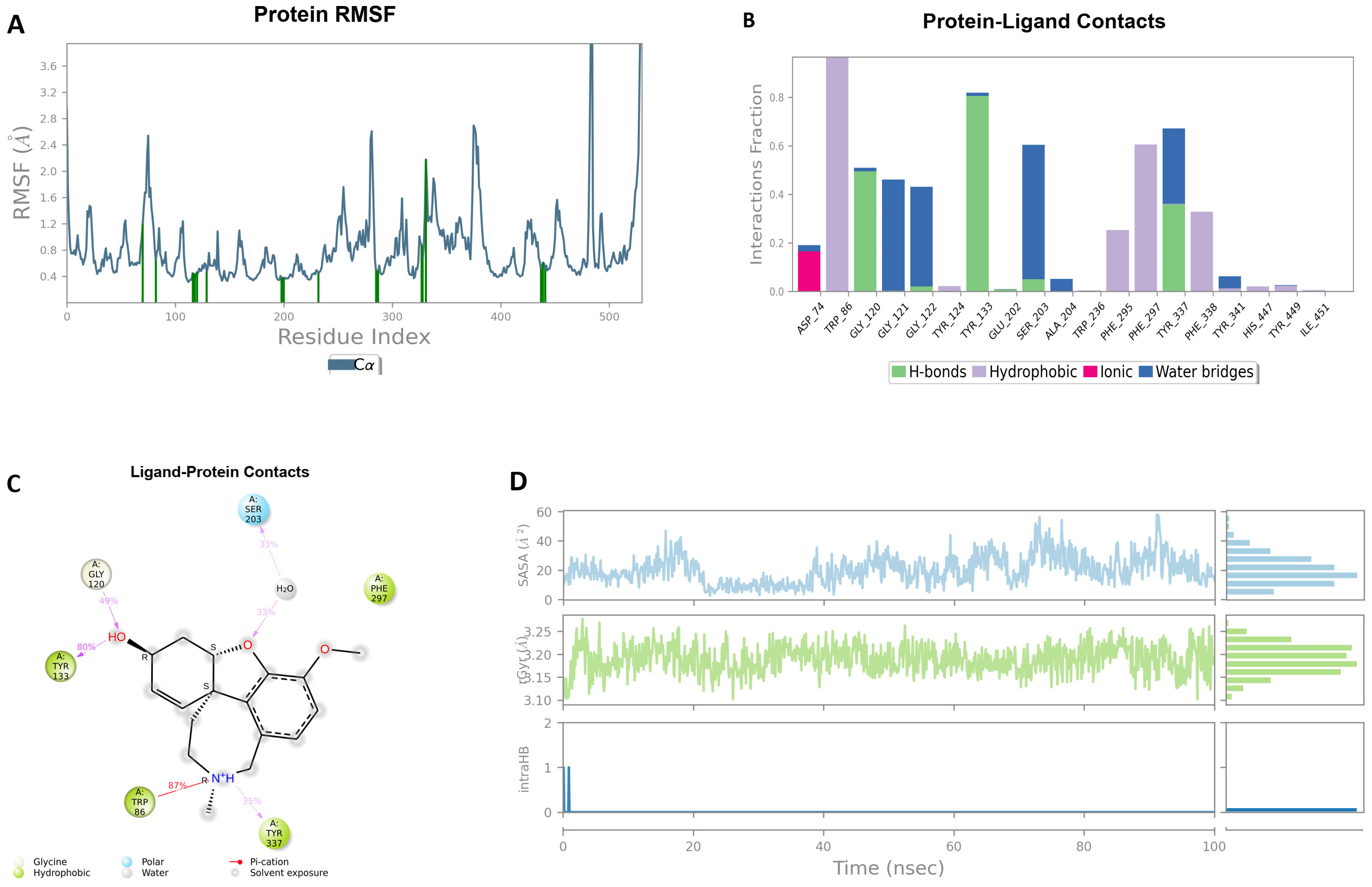

Supplement: S3 Fig — (TIF) [file pone.0284994.s003.tif]

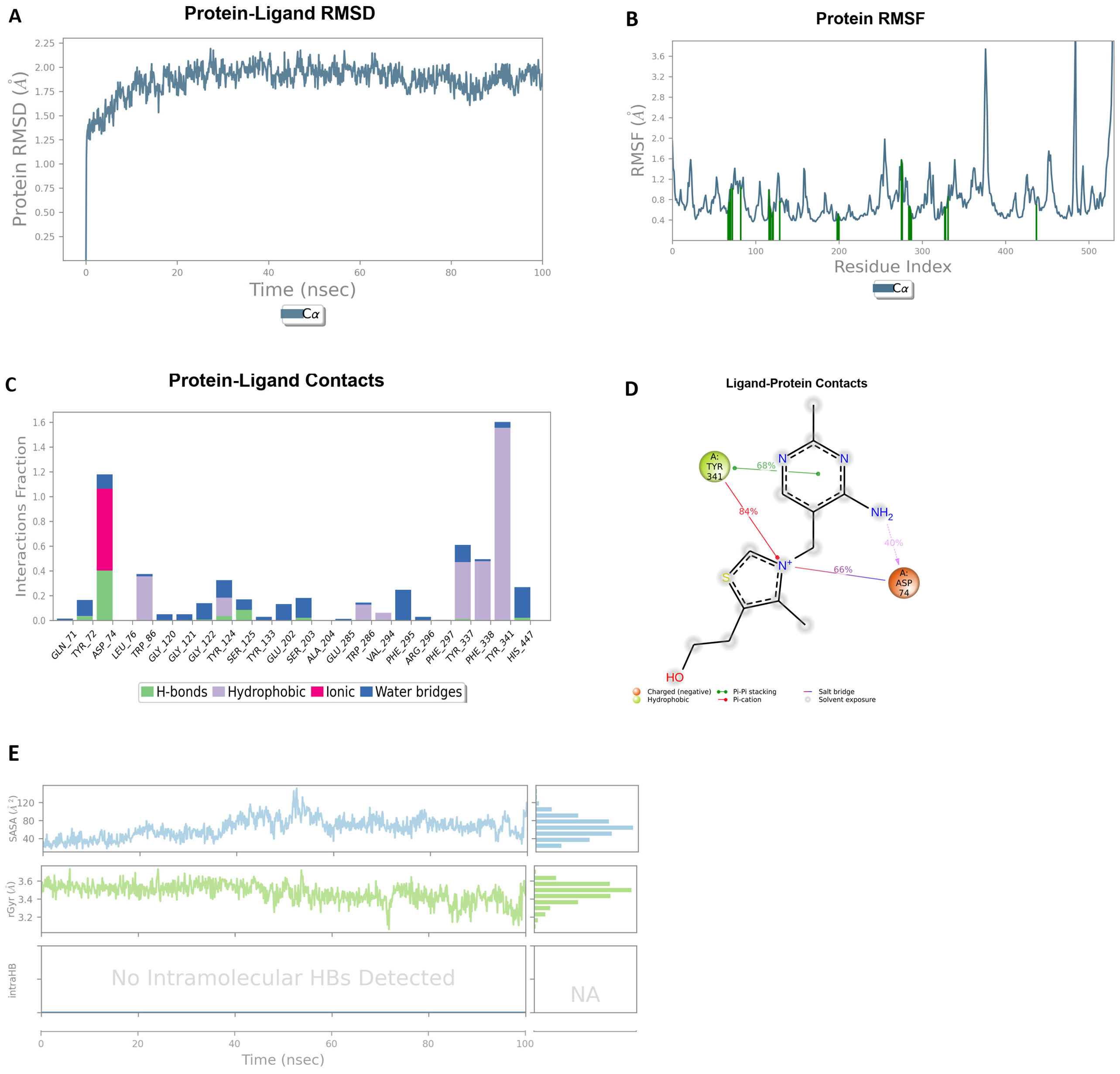

Supplement: S4 Fig — (TIF) [file pone.0284994.s004.tif]

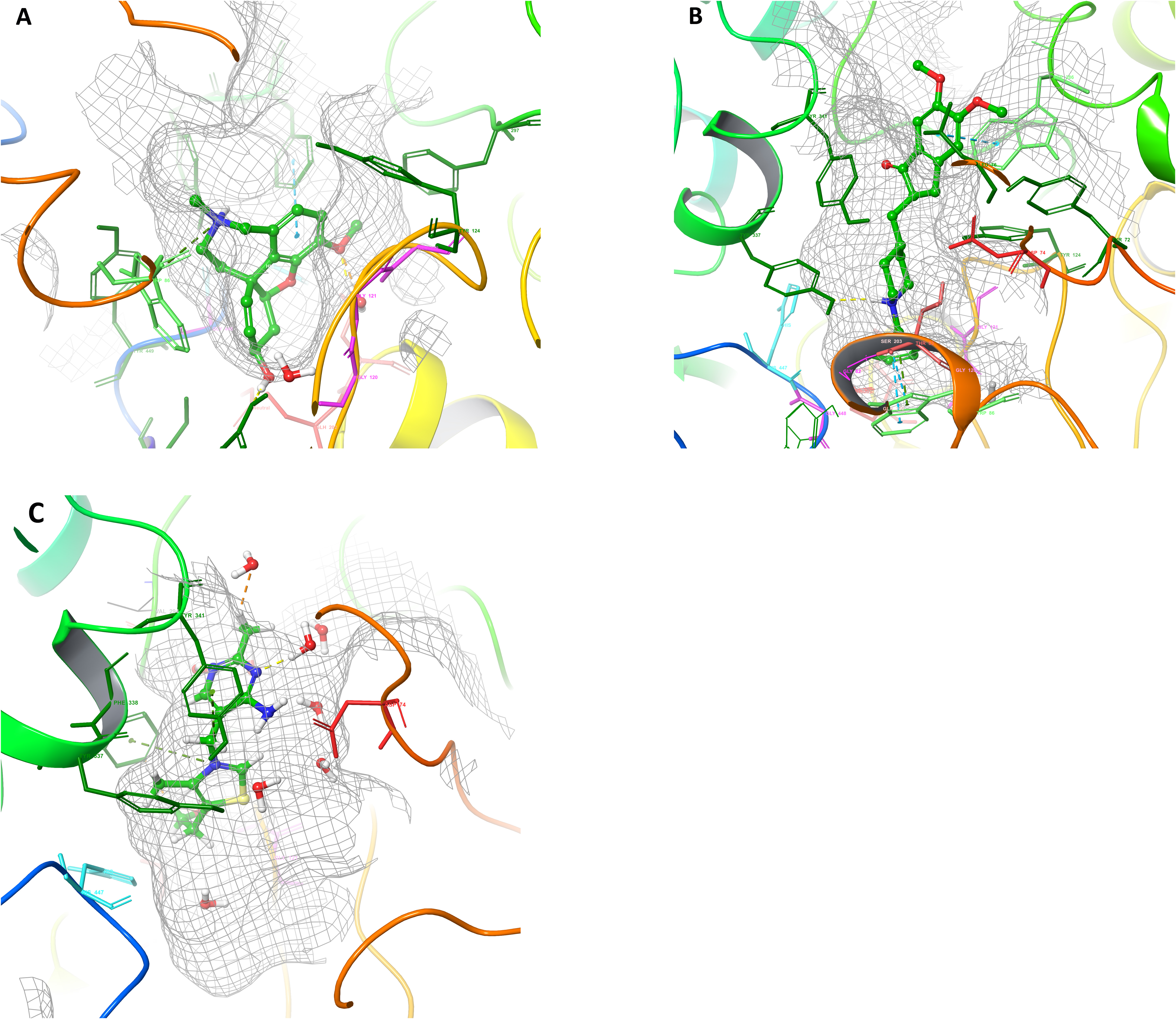

Supplement: S5 Fig — All three molecules maintain their stability within the binding pocket throughout the simulation trajectory. (TIF) [file pone.0284994.s005.tif]

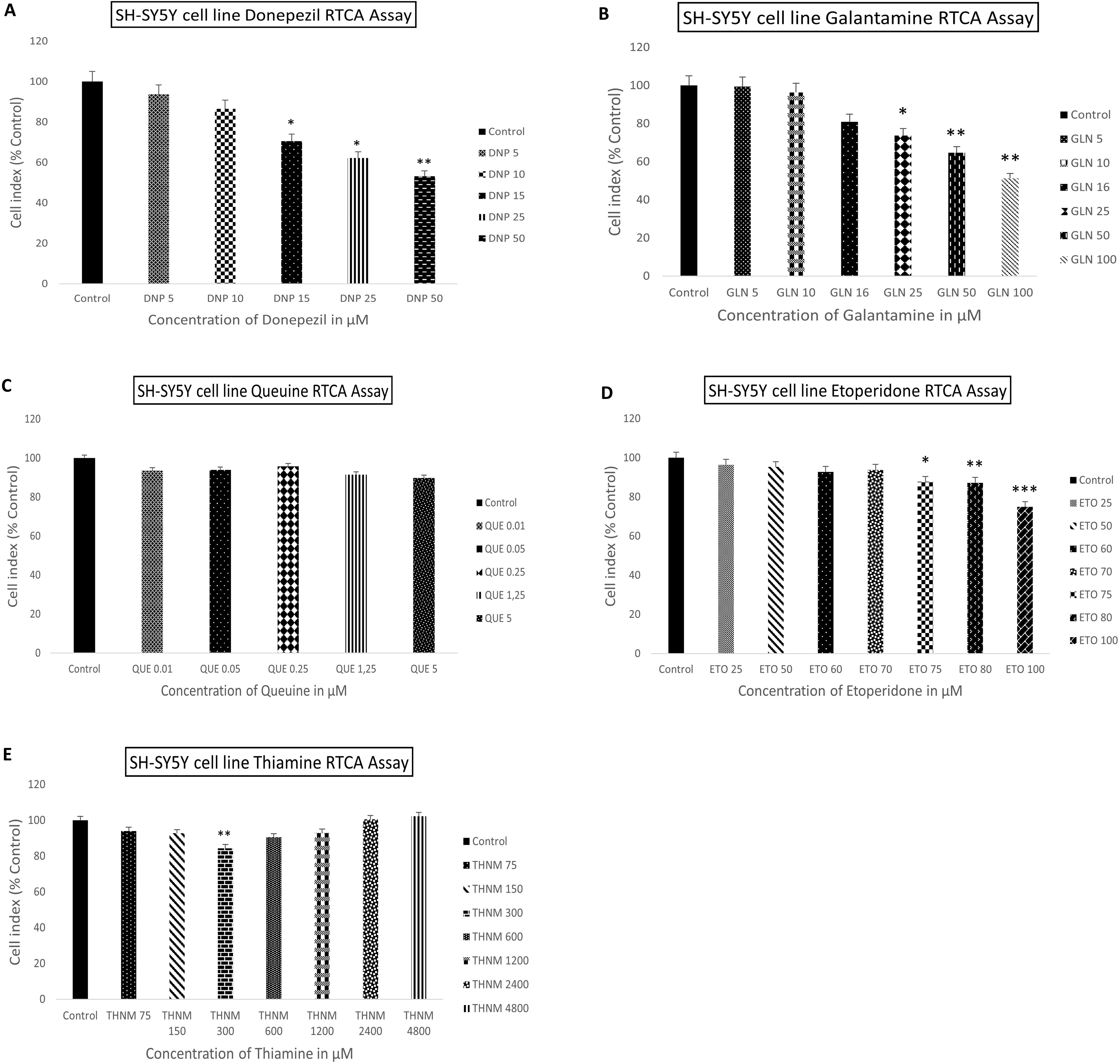

Supplement: S6 Fig — The results represent the average ± SEM of the results from each experiment, which were repeated three times at different times with the same concentration ranges. Compared to the control, *P\0.05, **P\0.01, ***P\0.001. (TIF) [file pone.0284994.s006.tif]
